# Towards Functional Insect Feeds: Agri-Food By-Products Enriched with Post-Distillation Residues of Medicinal Aromatic Plants in *Tenebrio molitor* (Coleoptera: Tenebrionidae) Breeding

**DOI:** 10.3390/antiox11010068

**Published:** 2021-12-28

**Authors:** Stefanos S. Andreadis, Nikolas Panteli, Maria Mastoraki, Eleftheria Rizou, Vassilia Stefanou, Sofia Tzentilasvili, Eirini Sarrou, Stavros Chatzifotis, Nikos Krigas, Efthimia Antonopoulou

**Affiliations:** 1 Institute of Plant Breeding and Genetic Resources, Hellenic Agricultural Organization—Demeter, 57001 Thermi, Greece; elef.rz@gmail.com (E.R.); esarroy@gmail.com (E.S.); nikoskrigas@gmail.com (N.K.); 2 Department of Zoology, School of Biology, Aristotle University of Thessaloniki, 54124 Thessaloniki, Greece; nkpanteli@bio.auth.gr (N.P.); mmastora@bio.auth.gr (M.M.); vasiliastefanou@hotmail.com (V.S.); tzentila@bio.auth.gr (S.T.); 3 Institute of Marine Biology, Biotechnology and Aquaculture, Hellenic Centre for Marine Research, Gournes Pediados, 71003 Heraklion, Greece; stavros@hcmr.gr

**Keywords:** circular economy, sustainability, essential oils, MAPs, yellow mealworm, edible insects

## Abstract

Sustainability, circular economy and alternative production systems are urgent imperatives for humanity and animal husbandry. Unless wasted, agri-food by-products can offer a promising source of high value. We evaluated the effect of rice bran (RB), corncob (CC), potato peels (PP), solid biogas residues (BR), and olive-oil processing residuals (OR), as alternative substrates to wheat bran (WB as control), on the growth and nutritional value of *Tenebrio molitor* during its breeding for animal feeds and/or human consumption. Innovation-wise, we further investigated the substrate supplementation (0, 10, 20%) with post-distillation residues of Mediterranean aromatic-medicinal plants (MAPs: lavender, Greek oregano, rosemary, olive; 1:1:1:1 ratio). *Tenebrio molitor* larvae (TML) were reared in all the studied substrates, and TML and diets’ proximate and fatty acid compositions as well as total phenol and flavonoid content and antioxidant potential were assessed using standard procedures. After statistical analysis of correlations, we observed that CC promoted oviposition and progeny survival; larval weight and dry matter were positively affected mainly by dietary energy and fat content; number of TML and/or larval weight increased using 10% MAPs inclusion in WB, RB and OR or RB, OR, BR and PP, respectively, which did not affect protein content; TML fatty acid composition decreased the content of saturated ones and increased that of mono-unsaturated ones; MAPs residues had an apparent favorable impact on total phenolic content and antioxidant activity of each substrate, with RB displaying the highest capacity and content. These findings indicate that alternative substrates can be exploited and their enrichment with natural phenolics is able to influence *T. molitor* growth, offering highly beneficial and nutritional value.

## 1. Introduction

To date, the rapid increase in demand for animal protein is driven by the continuous increase of world population, thus forcing consecutively the expansion of livestock industry [1]. However, livestock production is regarded as one of the major detrimental sectors of rural development in environmental terms, contributing to ecosystem degradation and disruption of biodiversity conservation [2]. Deforestation, landscape conversion to cropland, degradation and desertification of grasslands and pasture areas as well as water resource depletion are an immense reflection of livestock extensive requirements in arable land and fresh water [2,3]. Consequently, such anthropogenic environmental alterations trigger the onset of a sequential loss of species diversity [4]. Furthermore, soils, waters and atmosphere are dramatically burdened by conventional animal production. The constant discharge of high pollutant loads, including nutrients, organic matter, pathogens, heavy metals, antibiotics and drug residues, in surrounding soils and aquatic environments adversely affects ecosystem services [5], while livestock-produced greenhouse gases, including carbon dioxide, nitrous oxide and methane are among the greatest contributors to climate change [6].

The 17 Sustainable Development Goals (SDG) adopted by the United Nations General Assembly in 2015 aim to address the adverse effects of societal challenges, including poverty, climate change and environmental pollution, and are envisaged to ensure a more sustainable future for humanity, living species and the planet [7]. Among the targets of SDG 2 are the universal access to safe, nutritious and sufficient food and the transition to sustainable food production systems, which simultaneously falls within the target of SDG 12 for sustainable management and efficient usage of natural resources [7]. In this framework, compared to conventional livestock, insects’ rearing for mass consumption may mediate the reduction of ecological footprint of food production and mitigate climate change due to the lower greenhouse gas emissions and requirements in natural resources [8]. To date, insects are currently emerging as a potential sustainable source of protein in modern western societies; however, many insects have been consumed for ages as part of the daily human diet across many countries in East Asia, Africa, Central and South America [9]. Nutritional composition of insects varies depending on species, life stage, diet, etc. [10], but most edible insects provide sufficient energy, protein and fat content with well-balanced amino acid and fatty acid profiles and are a good source of micronutrients such as magnesium, phosphorous, biotin and riboflavin [8,11]. Insects are advantageous for their high feed conversion efficiency owned to their poikilothermic nature, which implies that body temperature regulation relies on the environment rather than the feed energy [12,13]. In this way, insects may be reared on different substrates including organic, industrial and agricultural waste streams, efficiently converting them into high-quality protein (nutritious biomass) [11,13]. Valorization of organic by-products for insect rearing aligns perfectly with circular economy principles, such as recycling and reuse of materials or wastes within extant production systems, thus substantially reducing waste release and minimizing their adverse impact on human health and the environment [14].

The potential utilization of agri-food waste and by-products in feed production resides to a large extent in the phytochemicals that are abundant in them and can be recovered as functional compounds [15]. Phytochemicals, such as phenols, flavonoids, essential oils and antioxidants, are bioactive compounds which are widely distributed in various parts of plants, vegetables and fruits, and they are able to prevent oxidative stress caused by ROS (Reactive Oxygen Species); therefore, they are well recognized for their beneficial effects on animal and human health [16,17]. Selection of edible insect species that are suitable for mass rearing is usually based on size, nutritional value, multivoltinism, sociability, safety, epidemic tendencies, reproductive and survival ability, and marketability [18]. In this respect, *Tenebrio molitor* L. (Coleoptera: Tenebrionidae), widely known as yellow mealworm, is one of the most important commercial edible insects with high reproduction rate, and is especially valued for its protein content [19]. Recently, *T. molitor* has been included among the insect species that are approved for use as ingredients in fish feed [20]; in addition, this was the first insect species to be characterized by the EFSA [21] as safe for human consumption in the form of dried mealworms or powder. *Tenebrio molitor* larvae (TML) are typically fed with a substrate of wheat bran supplemented with vegetables, fruits and/or protein sources [22]. However, recent studies have indicated that *T. molitor* can be fed with organic wastes converting them into valuable biomass of high nutritional value [23], thus suggesting that several wastes from production, processing and consumption of agricultural products could potentially be used as alternative feed sources during insect farming [24].

Among agricultural by-products, rice bran is an industrial by-product which is massively produced worldwide during grain processing. Although rice bran is considered to exert health-related benefits based on bioactive compounds, its utilization in food industry is still compromised due to instability or poor suitability issues, oxidation sensitivity, and extant anti-nutritional factors potentially associated with toxic results when consumed by humans. As a result, rice bran is either discarded as waste or used exclusively as feed [25,26]. Potato peels represent another by-product produced in large amounts by food-processing industries as well as an everyday life waste at homes and restaurants which are mainly discarded as zero-value wastes. However, potato peel wastes may intensify the environmental pollution adding high organic matter; therefore, an essential eco-friendly solution is needed in this direction [27]. A valuable alternative is the reuse of potato peels for feed replacement as nutrient-rich sources with high polyphenol content [28]. In addition, several agricultural by-products are derived from the processing of olives to produce the acclaimed olive oil, rendering this sector as one of the most economically important agro-industries worldwide. These olive oil residues can also be considered for further utilization and be used as feedstock [29]. Corn cob is another agricultural by-product with significant potential and functional properties that is often used as feed in livestock systems, but a great fraction is wasted, ending up in landfills [30]. Interestingly, corn cob contains 60% insoluble dietary fiber, with cellulose being the major constituent, and is a useful source of non-essential proteins and minerals (phosphorus, potassium and manganese), carotenoids (β-carotene, zeaxanthin and lutein) and phenolics with antioxidant properties [31]. Residual agricultural residues are often biodegraded to produce biogas. Solid biogas residue is the solid fraction that remains after anaerobic digestion of organic substrates by a series of microbial groups in the course of successive hydrolytic, acidogenic, acetogenic, and methanogenic processes [32], often including in Greece various materials such as corn straw, livestock manure as well as solid and liquid agro-industrial residues such as cheese whey [33]. On the other hand, during the last two decades, post-distillation residues (solid residues, wastewater, hydrolates) of medicinal aromatic plants (MAPs) from the essential oils market are known to be valuable sources of phenolic compounds of interest [34]. In addition, essential oils have been previously indicated as a natural source for feed additives with potent antioxidant capacity and strong inhibitory effects on pathogens growth [35,36,37]. It is worth mentioning that the ratio between the essential oils production and the plant biomass processed is very low, thus generating large amounts of wasted by-products [34]. To date, alternative substrates such as brewery spent grains [38], cereal products [39] or a mixture of by-products [40] and linseed-based substrates [41] are being evaluated in insect rearing.

In the frame of sustainability and circular economy in animal rearing for food and feed, the aim of the present study was to evaluate comprehensively the effect of several agri-food by-products or wastes as alternative substitutes of wheat bran on growth, nutritional value and beneficial content of *Tenebrio molitor*, an economically important insect for animal feeds and human consumption. Innovation-wise, we investigated for the first time herein the potential of optimization of these rearing substrates through supplementation with post-distillation residues of Mediterranean MAPs, thus coupling the reused wasted agricultural by-products (alternative substrate and MAPs processing residues).

## 2. Materials and Methods

### 2.1. Insect Colony

All insects used in this study derived from a stock colony that was maintained at the Entomology Lab of the Institute of Plant Breeding and Genetic Resources (IPGRB) of the Hellenic Agricultural Organization Demeter (Thermi, Greece). The colony was kept at 25 ± 1 °C, 60% relative humidity, under a 8:16 h light:dark photoperiod. Approximately 1000 adults of *T. molitor* were placed in transparent plastic trays (55 × 35 × 15 cm) with 1.5 kg of wheat bran as food source and oviposition substrate. After one week, adults were removed and transferred to a new tray with fresh substrate, whereas newly hatched larvae remained in the plastic tray for a period of approximately three months. When larvae reached pupation, they were sexed and placed separately into smaller plastic trays (20 × 11 × 4 cm) using featherweight soft forceps (BioQuip Products, Rancho Dominguez, CA, USA). Newly emerged virgin adults (one-two days old) were used for the bioassays. Both adults and larvae were provided with slices of fresh carrots twice a week as a water source.

### 2.2. Diet Preparation

The experimental treatments consisted of six different by-products used as substrates: wheat bran (WB) used as control, rice bran (RB), potato peel (PP) (dried at 50 °C for 24 h until constant weight), corn cob (CC), solid biogas residues (BR) and partially degraded olive oil processing plant residues (OR), while each substrate was supplemented with 0, 10% or 20% essential oils distillation residues (dried drogue) of lavender (*Lavandula angustifolia* Mill.), rosemary (*Salvia rosmarinus* Spenn.), Greek oregano (*Origanum vulgare* L. subsp. *hirtum* (Link) A. Terrac.), and olive-cake (*Olea europaea* L. subsp. *europaea*) at 1:1:1:1 ratio. The post-distillation dried residues of medicinal and aromatic plants were provided from IPGRB after the distillation of plant materials’ essential oils in Clevenger-type apparatus, and air-drying of the obtained plant biomass in 40 °C until constant weight in the oven. All post-distillation residues as well as dried potato peels and corn cob were pulverized in a mill before use (Ceccato M3, Ceccato Olindo, San Giorgio delle Pertiche PD, Italy).

### 2.3. Experimental Design

One pair of adults was introduced into a plastic cylindrical cup (5 cm in diameter, 8 cm in height) that was covered with a lid of fine mesh nylon on the top and left for reproduction and oviposition for a period of two weeks. Each cup was filled with 30 g of each feeding substrate, pure or supplemented with 10 and 20% essential oils distillation residues, using different cups for each treatment. Adult beetles left with 30 g of each substrate and/or supplemented with 10 and 20% essential oil distillation residues for two weeks for reproduction and oviposition. In all treatments, adults were provided with a slice of fresh carrot twice a week. After two weeks, adults were removed together with the carrot slides. For all bioassays, cups remained for an additional period of 12 weeks at the same conditions as described above allowing for colony growth. After this interval, the cups were opened and TML of each cup were separated from the feeding substrate, counted and weighed as a group to calculate the total larval fresh weight produced. Afterwards, TML were fasted for 24 h in order to evacuate their gastrointestinal tract from residual food, stored at −18 °C, dried using a freeze dryer (Freeze-dryer Alpha 1-2 LD plus, Christ, Osterode, Germany), and pulverized into a fine insect-powder that was kept at 4 °C until further use.

### 2.4. Proximate Composition

Insects’ as well as by-substrates’ proximate composition were assessed according to AOAC [42]. Moisture and dry matter were determined by dehydrating the samples at 90 °C until constant weight, while ash was calculated after incineration at 700 °C for 7 h. Crude fat was determined according to Folch et al. [43] using chloroform-methanol-BHT (2:1 *v/v* + 0.01% *w/v* BHT) extraction and energy content was measured using a bomb calorimeter (6300, Parr Instrument Company, St. Moline, IL, USA). Moreover, crude protein was determined using a nitrogen analyzer (FP-528, Leco corporation, St. Joseph, MI, USA) according to Dumas’s method. Two nitrogen-to-protein conversion factors (Kp) were used for the different substates’ protein content determination, i.e., Kp = 6.31 for WB [44] and Kp = 6.25 for PP [44,45], RB [46], CC [47], BR and OR. For the insect larvae, two different protein conversion factors were used due to the presence of non-protein nitrogen in compounds such as chitin and nucleic acids, i.e., the conventional Kp = 6.25 and Kp = 4.76, respectively, as proposed by Janssen et al. [48] to facilitate comparisons with other studies. Moreover, fiber and nitrogen-free extract (NFE%) were calculated by the following formula: NFE = 100% − (% crude protein + % lipid content + % moisture + % ash). All nutritional analyses were performed in triplicate for each sample.

### 2.5. GC-FID Analysis

Fatty acid composition was estimated by gas chromatography. At first, fatty acid methyl esters (FAMEs) were prepared according to AOCS [49]. FAMEs were then analyzed using a Shimadzu GC-2010 gas chromatograph (Shimadzu Corporation, Kyoto, Japan), equipped with a flame-ionization detector (GC-FID) and a SP-2330 capillary column (30 m × 0.25 mm i.d. × 0.20 μm film thickness) (Supelco Inc., Bellefonte, PA, USA). Helium was used as carrier gas at 2 mL/min constant flow; the split ratio was 1:50 and the injected volume 1.0 μL. The thermal gradient was 100 to 160 °C at 10 °C min^−1^, 160 to 220 °C at 3 °C min^−1^ and kept for 5 min, and lastly 220 to 250 °C at 10 °C min^−1^ and kept for 5 min. The injector and detector temperature were maintained at 260 °C and 280 °C, respectively. Fatty acids were identified by comparison with a known standard mixture of 37 key fatty acid methyl esters (Supelco 37 Component FAMΕ Mix, Sigma-Aldrich, Overijse, Belgium). Fatty acid methyl ester contents were expressed as a percentage (%) of total FAMEs basis.

### 2.6. Total Phenol and Flavonoid Content and Antioxidant Potential

In order to estimate total phenol and flavonoid content and antioxidant potential, three biological replicates/extractions of pulverized larvae and/or substrate (dried plant/fruit derived tissues) were selected for each treatment, since three biological replicates are considered an adequate size to detect alteration in dietary challenges [50]. Samples of 200 mg of freeze-dried larvae were mixed with 4 mL 80% methanol and 200 mg of dried plant/fruit derived substrate tissue with 8 mL 80% methanol into 15 mL falcon tube. The samples and solvent were sonicated for 20 min at room temperature and the extraction proceeded overnight at 4 °C. The resulting solutions where centrifuged at 10,000 rpm and the supernatant solution was used for the assays described below.

Total phenol content of methanolic extracts was determined with Folin-Ciocalteu reagent using gallic acid as a standard as described by Scalbert et al. [51] using a HITACHI, U-1900 UV-Vis ratio beam spectrophotometer (Hitachi High Technologies America, Inc., Schaumburg, IL, USA) for the measurements of absorbance. Total phenolic content was expressed in mg of gallic acid g^−1^ of tissue (larvae or substrate) dry weight (DW) using gallic acid as standard compound for calibration of curve linearity for concentration range 25–500 ppm (y = 0.0022x − 0.003, R^2^ = 0.9972). Total flavonoid content was determined colorimetrically as described by Zhisen et al. [52] and catechin was used as standard compound for the quantification of total flavonoids with calibration of curve linearity for concentration range 1–200 ppm (y = 0.0029x − 0.0064, R^2^ = 0.9989); values were expressed in mg of catechin g^−1^ of tissue (larvae or substrate) dry weight (DW). All measurements were conducted in triplicate.

As for the antioxidant activity, the Ferric Reducing Antioxidant Power (FRAP) was determined using a freshly prepared solution (0.3 M acetate buffer, pH 3.6), 10 mM TPTZ, 20 mM FeCl_3_·6H_2_O and 0.05 mL of methanolic extract, as previously described by Benzie and Strain [53].

Scavenging activity of the methanolic extracts was determined using 2,2′-azino-bis(3-ethylbenzothiazoline-6-sulfonate) (7 mM) reacting with K_2_S_2_O_8_ (2.45 mM) to a water solution as described by Re et al. [54]. All samples were measured in triplicate and the antioxidant activity was expressed in mg Trolox g^−1^ DW.

### 2.7. Statistical Analysis

Five replicates were used for the statistical analysis of the developmental characteristics of larvae. All samples for biochemical analysis were analyzed in triplicate and the results were expressed as mean values. The data were analyzed with Analysis of Variance (ANOVA), using the statistical package SPSS 11 17.0 (SPSS Inc., Chicago, IL, USA). Comparisons of means were accomplished with the Tukey’s test and standard error (S.E) was used to establish significant differences. The statistical significance in all hypotheses testing procedures was predetermined at *p* < 0.05. Correlation coefficients to determine the relationship between variables were calculated using Pearson Product Moment. Furthermore, a principal components analysis (PCA) was performed on total phenol and flavonoid content as well as antioxidant potential data through the FactoMineR package in the R programming environment to assess correlation patterns between different substrates and *T. molitor*.

## 3. Results

### 3.1. Alternative Substrates’ Composition

The proximate composition of the six experimental by-products used as alternative substrates of typical wheat bran is presented in Table 1. Dry matter of these substrates ranged from 78.1% to 93.5%. Crude protein was higher in the BR (22–25.3%), followed by WB (19.7–21.5%), and the lowest protein content was observed in CC (5.9–7.3%). Energy content was lower in the OR (5.8–8.6 MJ/kg), higher in RB (21.8–22.6 MJ/kg) and the rest of the substrates exhibited similar content (14.4–19.7 MJ/kg). Greatest differences were observed in the ash and fat contents. Rice bran (RB) had the highest fat content (19.1–20.9%) followed by WB (5.2–6.1%). Ash content was extremely high in OR (62.7–70.7%), followed by BR (24.9–27.3%). CC and PP had very low protein, fat, and ash content. The addition of MAPs generally increased the fat and energy content of the substrates and decreased the protein content (*p* < 0.05).

### 3.2. Growth Performance

Using the basic substrates, the number of TML grown in CC (48.6 ± 9.7) was significantly higher than that recorded in OR and BR (11.8–12.0, *p* < 0.05; Table 2). Addition of 10% MAPs significantly increased the number of TML grown in WB, RB and OR by an average of 122%, 124% and 197%, respectively (*p* < 0.05). Further supplementation of MAPs (20%) did not significantly alter the number of TML. The highest total dry larval weight was recorded for WB in all levels of MAPs inclusion (*p* < 0.05; Table 2). The incorporation of 10% MAPs increased total larval weights in RB, OR, BR and PP by an average of 269%, 531%, 435% and 309%, respectively (*p* < 0.05). Finally, further increase of MAPs (20%) did not alter significantly the total larval weight. Concerning the total larval weight on a dry-matter basis, the highest one was recorded for wheat bran (*p* < 0.05), which differed significantly from the rest of the substrates (Table 2). Similar results were obtained after the addition of 10% of MAPs. More specifically, addition of 10% MAPs increased total larval weight of all treatments except that of CC; however, only in the case of RB, PP, OR and BR this increase was significant (*p* < 0.05). Finally, further increase of MAPs (20%) did not alter significantly the total larval weight. Specifically, as in the case of the number of TML, total larval weight after the addition of MAPs (20%) was slightly lower in all treatments except that of OR and BR.

### 3.3. TML Proximate Composition

The proximate composition of TML fed with different substrates is presented in Table 3. Without the addition of MAPs, TML protein content was not affected by the different substrates. Dry matter was significantly higher in TML fed with PP (45.1 ± 1.3%) followed by RB and WB (39.3–39.5%; *p* < 0.05). Fat content was significantly higher in TML fed with RB (31.3 ± 0.3%; *p* < 0.05). Fat content of the treatments was similar (23.9–25.2% *p* > 0.05), except the fat content of TML fed with CC, which was slightly lower (16.5 ± 1.1%; *p* > 0.05). Ash content appeared to be significantly higher in TML fed with BR and PP (10.2–10.3%; *p* < 0.05). Moreover, fiber and NFE was significantly higher in TML fed with CC (27.5 ± 1.6%) compared to larvae fed RB, OR, BR and PP (8.5–13.2%; *p* < 0.05).

The incorporation of MAPs in the substrates did not affect protein content in larvae fed with WB, RB and CC; however, it significantly lowered the protein content of TML fed with PP and OR (*p* < 0.05). Fat content was significantly increased by the MAPs addition in the PP treatment and was significantly lowered in the OR treatment. An inverse effect was observed in TML’s dry matter, where the incorporation of MAPs significantly decreased dry matter content in TML fed with PP (*p* < 0.05). Ash content was significantly lower in TML fed with PP and BR supplemented with MAPs, compared to the basic substrate without the addition of MAPs (*p* < 0.05). Finally, the introduction of MAPs in the substrates used for the rearing of TML led to increased fiber and NFE content in OR and PP treatments. Overall, the addition of MAPs seems to affect the proximate composition of the TML fed with OR, BR and PP. The two-way ANOVA was used to reveal the interaction between the different types of substrates and the addition of MAPs.

### 3.4. Effect of Substrates’ Proximate Composition on Growth and TML Nutrient Content

Table 4 includes the detected correlations between the proximate composition of the different experimental by-products used as alternative substrates and the growth and/or the nutritional value of TML. The protein and dry matter content of the substrates did not correlate with the number of TML produced, the total larval weight or their nutritional value (*p* > 0.05), except for fiber and NFE content, where we observed a negative correlation with substrates protein (r = −0.598; *p* < 0.05). Substrates’ fat content had a moderately positive correlation with the total larval weight (r = 0.636; *p* < 0.01) and TML fat content (r = 0.684; *p* < 0.01) as well as a marginal correlation with TML dry matter (r = 0.496; *p* < 0.05), while it did not appear to affect the number of TML nor TML protein, ash, fiber and NFE content. The energy content of the substrates had a high positive correlation both with the number of TML (r = 0.727; *p* < 0.001) and total larval weight (r = 0.843; *p* < 0.001), and a moderate correlation with TML dry mass (r = 0.591; *p* < 0.01) and TML fat (r = 0.643; *p* < 0.01), while ash content correlated negatively with the number of TML (r = −0.682; *p* < 0.01) and total larval weight (r = −0.671; *p* < 0.01).

### 3.5. TML Fatty Acids Profile

The fatty acids profile of the ΤΜL reared on the by-products used as experimental substrates is presented on Table 5 and Figure S1. Without the addition of MAPs, the saturated fatty acids were significantly higher in TML fed with BR and PP (36.6% and 37%, respectively) and significantly lower in larvae fed with WB (18.9%; *p* < 0.05). The dominant saturated fatty acid was palmitic acid (C16:0). The mono-unsaturated fatty acids content was significantly higher in the TML fed with OR and WB (50.2% and 50.7%, respectively), driven by the significantly higher oleic acid (18:1, ω9) content of these TML (*p* < 0.05). The poly-unsaturated fatty acids content of the TML fed WB was significantly higher (29.1%; *p* < 0.05) followed by TML fed with WB (27.8%). Additionally, the omega-6 content followed the same pattern with PUFA content, due to the significantly higher linoleic acid (18:2, ω6) content of these TML (*p* < 0.001).

### 3.6. Total Phenol and Flavonoid Content and Antioxidant Potential

**Figure 1:**
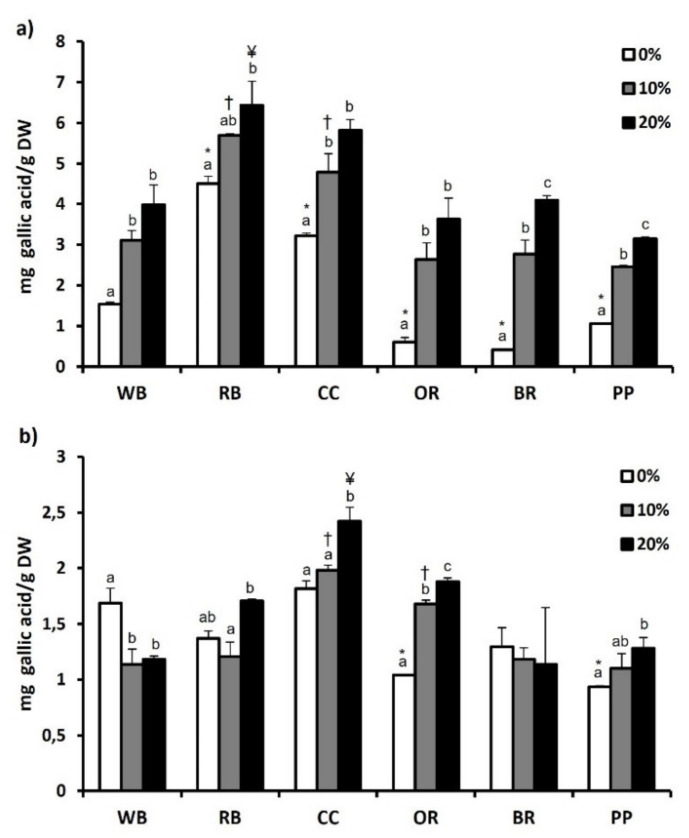
Total phenolic content of (**a**) agri-food substrates and (**b**) *Tenebrio molitor* larvae reared on the respective substrates (WB, Wheat bran; RB, Rice bran; CC, Corn cob; OR, Olive oil residues; BR, Biogas residues; PP, Potato peel). Different bar colors depict supplementation percentage of post-distillation residues of medicinal and aromatic plants (MAPs) in the substrates. Bars represent mean values of three replicates ± SE. Different letters depict significant differences regarding MAPs post-distillation residues supplementation in each substrate, while different symbols are used (*, †, ¥) to denote significant differences between substrates compared to the WB in the respective supplementation percentage of MAPs post-distillation residues (0%, 10%, 20%, respectively).

**Figure 2:**
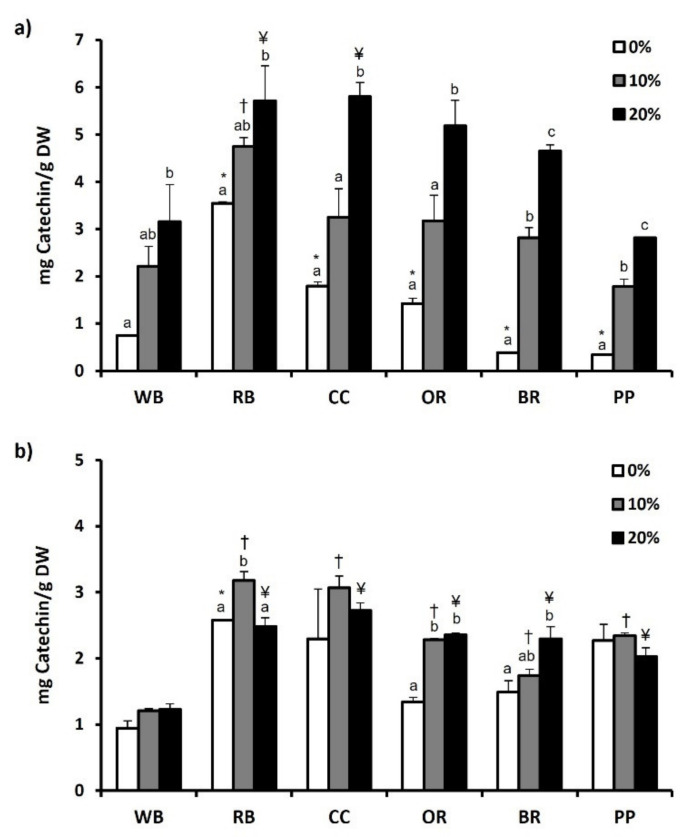
Total flavonoid content of (**a**) agri-food substrates and (**b**) *Tenebrio molitor* larvae reared on the respective substrates (WB, Wheat bran; RB, Rice bran; CC, Corn cob; OR, Olive oil residues; BR, Biogas residues; PP, Potato peel). Different bar colors depict supplementation percentage of post-distillation residues of medicinal and aromatic plants (MAPs) in the substrates. Bars represent mean values of three replicates ± SE. Different letters depict significant differences regarding MAPs post-distillation residues supplementation in each substrate, while different symbols are used (*, †, ¥) to denote significant differences between substrates compared to the WB in the respective supplementation percentage of MAPs post-distillation residues (0%, 10%, 20%, respectively).

The total phenol content of both experimental substrates and TML that were fed with them are depicted in Figure 1a,b, respectively, while total flavonoid content is presented in Figure 2a,b. Regarding substrates, RB showed significantly (*p* < 0.05) higher total phenol (4.50 ± 0.18 mg Gallic acid/g DW) and flavonoid content (3.54 ± 0.04 mg Catechin/g DW), compared to WB and all other substrates. The second highest total phenol (3.22 ± 0.07 mg Gallic acid/g DW) and flavonoid content (1.79 ± 0.09 mg Catechin/g DW) was observed in CC. In contrast, BR displayed the lowest total phenol content (0.41 ± 0.03 mg Gallic acid/g DW) (Figure 1a). Furthermore, total flavonoid content was significantly lower in BR and PP, compared to WB as well as RB, CC and OR (*p* < 0.01) (Figure 2a). Regarding *T. molitor*, RB, CC and BR substrates resulted in no significant changes in total phenols content compared to WB, while significant reduction was observed in OR and PP (Figure 1b). Among the substrates, RB favored the increase (*p* < 0.05) of total flavonoid content of *T. molitor* (Figure 2b).

The addition of MAPs in the substrates had as a result the increase of total phenols and flavonoids in all substrates, with the 20% supplementation showing a significant increase compared to 0%. However, this pattern was not found in TML fed with substrates supplemented with 10 or 20% of MAPs. In specific, 10% MAPs supplementation favored the increase of total phenols only in OR-reared TML, while 20% supplementation resulted in a significant increase in *T. molitor* reared on CC, PP and OR substrates (Figure 1b). Total flavonoid content of *T. molitor* was favored by 10% supplementation in RB substrate and by 20% supplementation in OR and BR substrates (Figure 2b).

**Figure 3:**
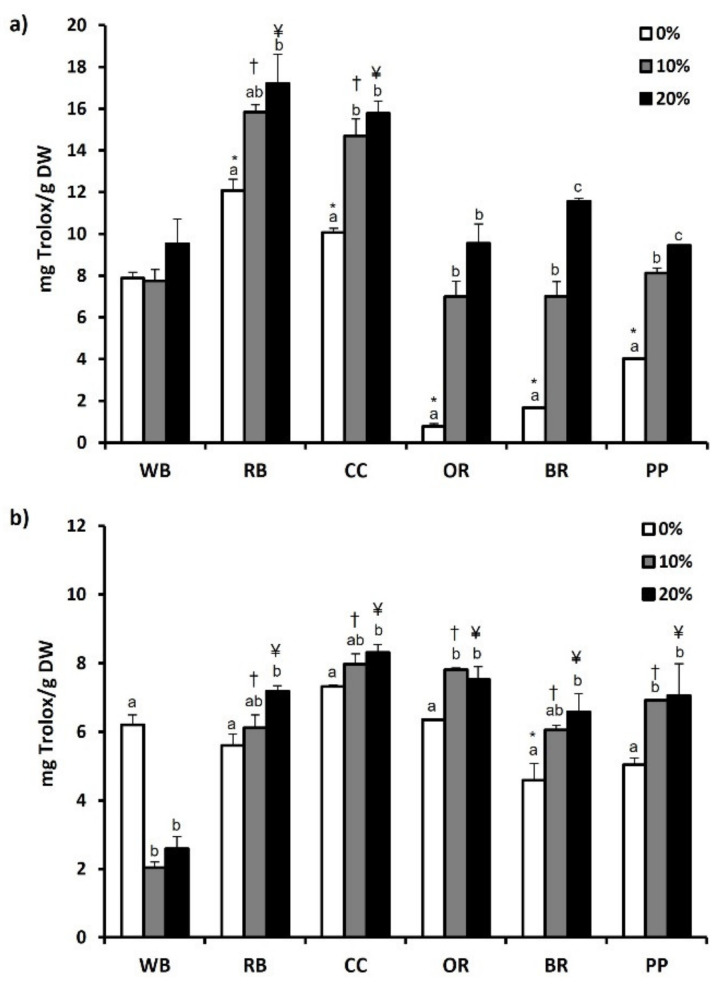
The antioxidant potential as determined by ABST assay of (**a**) agri-food substrates and (**b**) *Tenebrio molitor* larvae reared on the respective substrates (WB, Wheat bran; RB, Rice bran; CC, Corn cob; OR, Olive oil residues; BR, Biogas residues; PP, Potato peel). Different bar colors depict supplementation percentage of post-distillation residues of medicinal and aromatic plants (MAPs) in the substrates. Bars represent mean values of three replicates ± SE. Different letters depict significant differences regarding MAPs post-distillation residues supplementation in each substrate, while different symbols are used (*, †, ¥) to denote significant differences between substrates compared to the WB in the respective supplementation percentage of MAPs post-distillation residues (0%, 10%, 20%, respectively).

**Figure 4:**
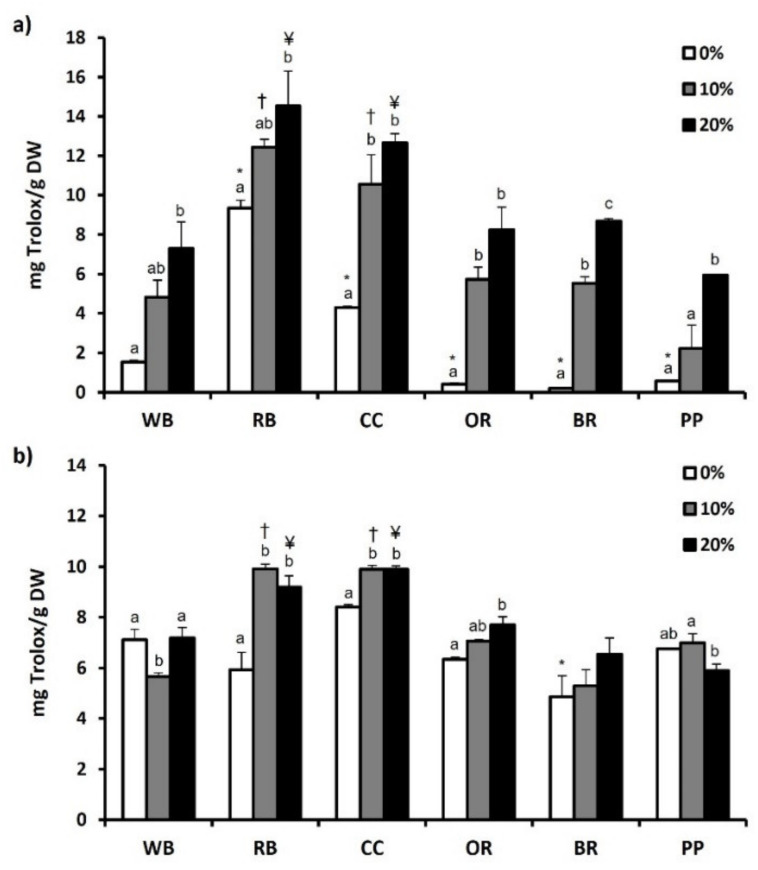
The antioxidant potential as determined by FRAP assay of (**a**) agri-food substrates and (**b**) *Tenebrio molitor* larvae reared on the respective substrates (WB, Wheat bran; RB, Rice bran; CC, Corn cob; OR, Olive oil residues; BR, Biogas residues; PP, Potato peel). Different bar colors depict supplementation percentage of post-distillation residues of medicinal and aromatic plants (MAPs) in the substrates. Bars represent mean values of three replicates ± SE. Different letters depict significant differences regarding MAPs post-distillation residues supplementation in each substrate, while different symbols are used (*, †, ¥) to denote significant differences between substrates compared to the WB in the respective supplementation percentage of MAPs post-distillation residues (0%, 10%, 20%, respectively).

Furthermore, antioxidant capacity of substrates and TML was evaluated using ABST and FRAP assay. Compared to WB and the other substrates, RB showcased significantly (*p* < 0.05) higher ABST (12.09 ± 0.55 mg Trolox/g DW) (Figure 3a) and FRAP (9.35 ± 0.39 mg Trolox/g DW) values (Figure 4a). CC displayed the second highest ABST (10.07 ± 0.21 mg Trolox/g DW) and FRAP (4.30 ± 0.05 mg Trolox/g DW) values. In contrast, BR displayed the lowest FRAP value (0.19 ± 0.02 mg Trolox/g DW) (Figure 4a), while both OR and BR showcased the significantly lowest ABST values (0.80 ± 0.12 mg Trolox/g DW and 1.69 ± 0.04 mg Trolox/g DW, respectively; *p* < 0.05). Regarding TML, BR led to a significant decrease in ABST and FRAP values, compared to WB (Figure 3b and Figure 4b, respectively). However, no significant differences were observed in the aforementioned values of TML reared in other experimental substrates. Following supplementation of MAPs, ABST and FRAP values increased significantly in all substrates compared to absence (0%) of MAPs. Similarly, 20% MAPs addition in the substrates favored the increase of both ABST and FRAP values in RB, CC, OR and PP. On the contrary, the antioxidant potential of TML reared on WB displayed a reduction in response to MAPs supplementation.

A correlation analysis was conducted to evaluate whether the total weight of TML was correlated with the number of TML, their content in phenols and flavonoids and their antioxidant potential (Table 6). The analysis was performed once for each substrate separately, and again enabling all substrates of our experimentation in order to produce more conclusive results (in case of diverse larvae behavior under each substrate fed). According to this analysis, total weight and number of TML were highly correlated under the substrate feeding WB (r = 0.915, *p* < 0.01), RB (r = 0.896, *p* < 0.01) and CC (r = 0.578, *p* < 0.01). The total weight of TML was not correlated with their total phenolic content in all tested substrates except in OR where positive correlation was observed (r = 0.91, *p* < 0.01). When TML were fed in OR and BR, significant correlations were observed between the total weight of TML and flavonoids (r = 0.911, *p* < 0.01, and r = 0.681, *p* < 0.05, respectively) which might also reflect to the positive correlations represented between the total weight of TML and ABTS (r = 0.916, *p* < 0.01, and r = 0.773, *p* < 0.05, respectively) and FRAP antioxidant potential (r = 0.786, *p* < 0.01 for olive residues).

Furthermore, we were able to identify positive correlations between substrates and TML antioxidant contents (Table 7). In specific total phenols content of substrate and TML were highly correlated in CC (r = 0.922, *p* < 0.01), OR (r = 0.955, *p* < 0.01) and PP (r = 0.69, *p* < 0.01) treatments. Moreover, positive correlations were observed in total flavonoid content of substrate and TML in OR (r = 0.819, *p* < 0.01) and BR (r = 0.841, *p* < 0.01). Regarding the antioxidant potential in all feeding substrates, positive correlations were observed, except WB and CC for ABTS assay, and WB, BR and PP for FRAP assay.

**Figure 5:**
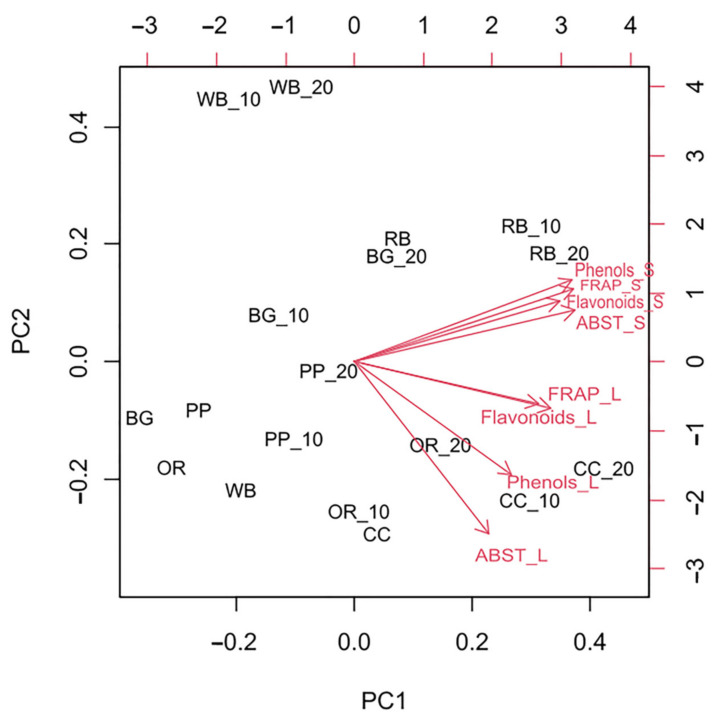
Principal Component Analysis (PCA) performed on total phenols and flavonoid content and antioxidant potential of substrates and *Tenebrio molitor* larvae reared on the respective substrates (WB, Wheat bran; RB, Rice bran; CC, Corn cob; OR, Olive oil residues; BR, Biogas residues; PP, Potato peel). Red vector arrows were included as predictors in the PCA construction.

According to PCA analysis (Figure 5), the first axis (PC1) explained 68.71% of the variance. Total phenols content and ABST values of substrate as well as total flavonoids content and FRAP values of both substrate and TML were positively correlated with PC1 scores. On the contrary, total phenols content and ABST values of TML were negatively correlated with scores on the second axis (PC2). The cumulative value of PC1 and PC2 was 83.45%.

## 4. Discussion

### 4.1. Growth of TML

Insect fecundity, oviposition and growth can be majorly affected by the diet composition, and numerous studies have evaluated different substrates for efficient mass production of *Tenebrio molitor*. In our study, higher numbers of oviposition and progeny survival, as expressed by the number of TML present at the end of the experimental period, was significantly higher when CC was used as a substrate compared to OR and BR which were the substrates with the lowest energy content. Nevertheless, the latter substrates were also the ones with the higher protein content. In insects, and specifically in *T. molitor*, diets with higher protein content have been reported to positively affect female reproduction rates and oviposition [55,56]. Previous studies [57] have reported a significant increase in the daily egg production of *T. molitor* in substrates with higher protein content and of similar energy content or higher reproduction rates of *T. molitor* in starch-rich substrates compared to protein-rich substrates with similar energy content [39]. Other studies have reported that the dietary protein content in tandem with the carbohydrate levels can influence survival and growth [58,59]. Herein, the number of surviving offspring was significantly affected by the energy and ash content, as the correlation showed. Despite the similar number of TML, WB resulted in significant higher larval weight compared to RB and CC, while PP led to the lowest larvae weight among them. These results could be attributed to the overall composition of the four substrates. A correlational analysis revealed that larval weight can be positively affected by dietary energy and fat levels and to a lesser extent by fiber and NFE levels. It is known that structural fiber, such as neutral detergent fiber and lignin, cannot be efficiently utilized by insects [60] and can negatively affect growth performance [61]. However, *T. molitor* has the ability to digest dietary fiber due to endogenous enzyme secretion and suitable intestinal microbiome composition [62]. Regarding the dietary protein to carbohydrate levels, lower *T. molitor* pupae weight have been reported when reared in substrates with low protein/high starch content compared to high protein/high starch and high protein/low starch [63]. In this study, the observed differences in larval weight could be explained by the documented protein/starch ratio for WB, RB, CC and PP (0.75, 0.51, 0.41 and 0.15, respectively [64,65,66,67].

As the addition of MAPs increased the energy and fat content of the diets and decreased the ash, an increase in the number of TML was observed when 10% of MAPs were incorporated in WB, RB and OR, while an increase in total larval weight was also detected when 10% of MAPs were included in RB, OR, BR and PP. In the substrates with lower nutritional value, such as BR and OR with high ash content or PP with very low-fat content, the addition of MAPs provided extra macronutrients which probably gave the TML the opportunity to choose between the feed particles in the substrate mixture to fulfill their nutritional needs more adequately. Further increase in the MAPs inclusion did not affect TML number and weight in our study possibly due to the antagonism for available nutrients (in nutrient-poor substrates) and/or due to increase in the presence of plant secondary metabolites negatively affecting growth performance. A possible explanation for this may be sought in the rich polyphenolic content known in many *Lamiaceae* plants; the latter can reduce beetle survival and growth rates in *Leptinotarsa decemlineata* and *Acanthoscelides obtectus* beetles, thus offering protections against pests [68,69].

### 4.2. Composition of TML

Larval dry matter was greatly affected by the dietary energy and fat content. The protein content of the TML in simple alternative substrates ranged between 50.7–59.5% (Kp = 6.25 and 38.6–45.3% Kp = 4.76) which was similar to other studies where TML were fed brewery spent grains [38], cereal products [39] or a mixture of by-products [40]. Despite the wide range of dietary protein (7.3–25.3%), TML were able to maintain similar protein content when fed with the different substrates and to efficiently utilize low quality substrates high in ash and fiber, thus accumulating a substantial amount of protein in their bodies. Especially since insect meals are marketed as a protein source, rich content is essential. In the literature, insect protein content has been correlated with the dietary protein [39], and dietary fat and fiber [70]. In this study, protein content did not correlate with the substrates’ proximate composition, leading to the assumption that TML adapted their substrate consumption to maximize nutrient intake regardless of the different dietary composition. An inclusion of 10% MAPs overall did not appear to affect protein content, but a further incorporation of MAPs generally reduced protein content.

Fat content was not affected by the addition of MAPs but was greatly influenced by the fat and energy content of the substrates. In agreement with our results, other studies, in which dietary fat content was reduced by 58%, observed a 28% decrease of fat content in *T. molitor*, 25% in *Zophobas morio* and 39% in *Alphitobius diaperinus* (both Coleoptera: Tenebrionidae; [63]). Despite the extremely low dietary fat content of some of the substrates used, TML herein were able to accumulate/synthesize fat and obtain fat content over seven times higher than that of dietary fat levels in the case of CC, 10 times higher in the case of BR or over 19 times higher than that of dietary fat in OR and PP. Insect fat has a very important role as it can prevent moisture loss and can provide energy in periods of high energy requirement such as growth, reproduction or prolonged starvation [71,72]. Generally, it is known that to obtain body fat, insects can utilize carbohydrates to synthesize fat [71].

### 4.3. Fatty Acids of TML

*Tenebrio molitor* larvae (TML) reared with all the alternative experimental substrates were rich in mono-and poly-unsaturated fatty acids. The fatty acid composition was greatly affected by the different rearing substrates. Without the use of MAPs, saturated fatty acids ranged from 18.9% to 37.0%, with palmitic acid having the highest share. These results agree with previously reported saturated fatty acid content of TML [73,74,75]. Mono-unsaturated fatty acids were the highest represented fatty acid group and ranged between 44 and 50.7%. Despite the significant differences in the TML fed the different experimental substrates, the variation was low. Consistent with other findings, oleic acid (C18:1) represented over 90% of the mono-unsaturated fatty acids of the larvae [73,74,75,76]. Poly-unsaturated fatty acids and omega-6 fatty acid content followed the same pattern due to the high linoleic acid content (C18:2 ω-6). Omega-3 content was extremely low and the only identified omega-3 fatty acid was α-linolenic acid (C18:3 ω-3) with an average content of 0.18% (data not shown). Generally, previous studies report a low C18:3 content in TML (0.10–1.5% [73,74,77]. However, insect fatty acid profile can be manipulated by the diet introduced, and farmed insect can be fortified with omega-3. For example, linseed-based substrates can enhance the C18:3 content of TML up to 12% [41], and brown algae and fish-offal substrate supplementation can enrich *Hermetia illucens* larvae with the beneficial eicosapentaenoic acid (C20:5 ω-3) [78,79] which is absent in most terrestrial insects [80].

In this study the supplementation of MAPs only improved TML fatty acid composition by generally decreasing saturated fatty acid content and increasing mono-unsaturated fatty acid content. Saturated fatty acids can cause hypercholesterolaemia, induce the build-up of vascular plaque, thus increasing the risk of thrombosis [81]. On the other hand, mono-unsaturated fatty acids can lower blood cholesterol and slow down the progression of plaque accumulation in the arteries [81].

### 4.4. Phenols-Flavonoids

Rearing substrate as dietary niche is a pivotal factor in insect development and breeding with an immense impact on nutritional composition as well as larval cycle length, size and weight [82]. Agri-food by-products are often characterized by high contents of bioactive compounds which may be exploited by feed industry as natural sources of functional ingredients for animal feeds [15]. Among the by-products used as substrate in the present study, rice bran displayed the highest antioxidant capacity and total phenol and flavonoid content, which are in alignment with previous results using methanolic rice bran extracts [83]. Rice bran (RB) is considered an important bio-resource due to its phytochemical profile and concomitant antioxidant activity, rich in bioactive non-nutrient compounds such as carotenoids, phenolics, flavonoids and alkaloids [84,85]. From the obtained results herein, it is clear that rice bran waste as a rearing substrate favored the flavonoids’ increase in *T. molitor*. Previous investigations in other insects indicated that flavonoids levels in the blue butterfly *Polyommatus icarus* are positively affected by the amounts of the consumed dietary flavonoids [86]. Degradation of dietary phenolics has been reported in some insect species, suggesting that utilization of these compounds may contribute to fitness improvement [87].

Similar to RB, CC may also be regarded as beneficial for reuse in feed production since it contains high phenolic and flavonoid contents as well as antioxidants such as p-coumaric acid and ferulic acid [88]. Herein, CC showcased higher total phenol and flavonoid content, and strong antioxidant capacity compared to WB that is traditionally used for livestock feed [89]. In contrast to the previously mentioned, lower flavonoid content was observed herein regarding PP which also showcased lower (rather average) gallic acid content. The latest is regarded as one of the main phenolic compounds in potato skin, highly attributing to its antioxidant and anti-inflammatory activities [90,91]. In comparison with our results, other studies have reported higher phenolic and flavonoids content in PP [92] than that found herein and stressed out the influence of extraction solvents used to obtain results. Nonetheless, variations in phenolic and flavonoid content between studies may be due to differences regarding a plethora of factors involved such as genotype, growing conditions, postharvest storage and/or experimental methods [83,89,90]. Previous investigations have indicated that phenolic compounds concentration in peel powder extracts differs depending on potato variety [90]. Furthermore, miscellaneous environmental factors such as temperature, oxygen and light may also exert significant influence on the physicochemical stability of phytochemicals [93]. Exposure of many polyphenolic compounds such as caffeic and gallic acid to high pH may induce formation of unstable quinone intermediates, thus leading to non-reversible chemical transformations and degradation. Such susceptibilities to pH vary between compounds due to structural differences such as the presence of phenolic-OH groups in gallic acid [94].

In this study the supplementation of essential oils distillation residues had an apparent favorable impact on total phenolic content and antioxidant activity of each substrate. Essential oils and aromatic compounds serve as a substantial source of phenolic compounds with potent antioxidant activity and may therefore have commercial applications in feed industry as sensory additives, flavoring agents and appetizing substances [35,95]. In addition, herbal crude extracts are widely used as natural food preservatives due to the inhibitory role of antioxidants in lipids oxidative degradation, thus improving food nutritional value [96,97].

Bioactive substances are also found in insects derived either from ingestion of plant materials and subsequent metabolic procedures or by de novo synthesis through the sclerotisation process [98]. In our study, the addition of MAPs post-distillation residues in RB, OR and BR exerted an ameliorating effect on total flavonoid content of *T. molitor*. Among plant phenolics, insects appear to absorb flavonoids more frequently due to the high sequestration and metabolism capacity for these specific compounds [98]. Nonetheless, sequestered amounts are highly determined by the flavonoid type in the feeding regime [87], thus explaining differentiations observed herein regarding total content of TML reared in various alternative substrates. Furthermore, dietary exposure to flavonoids and phenols has been previously reported to affect insect growth, depending on the compound type as well as insect species [99,100]. Thus, increase in total larval weights following supplementation of MAPs residues in RB, OR, BR and PP may indicate that substrate enrichment in phenolics is able to influence *T. molitor* growth. In addition, the beneficial effect of MAPs supplementation is reflected in the increased antioxidant activity of *T. molitor* reared in both RB and CC. However, it must be emphasized that usually the protein antioxidant capacity is not taken into consideration [101], and thus the potential total antioxidant activity of insects may be even greater.

## 5. Conclusions

In the frame of sustainability, agri-food by-products can be exploited by the feed industry as natural sources of functional ingredients for animal feeds or human consumption due to their high nutritional value and rich content of bioactive compounds with beneficial properties. With limited research on this subject to date, the study herein investigated, for the first-time, alternative substitutes of wheat bran in *Tenebrio molitor* breeding such as rice bran, corn cob, potato peels, solid biogas residues, and olive-oil processing residuals. These were investigated in respect to growth, nutritional value and beneficial content during *T. molitor* breeding, which is an economically important insect officially able to be exploited for animal feeds and/or human consumption. Innovation-wise, the study herein further investigated the substrate supplementation (0, 10 and 20%) with post-distillation residues of typical Mediterranean aromatic-medicinal plants (lavender, Greek oregano, rosemary, olive; 1:1:1:1 ratio), thus coupling the agri-by-products exploited sustainably in insect rearing. The results presented herein showcased that RB and CC are valuable alternative substrates for *T. molitor*. Furthermore, our study showed that the increase in total larval weights following supplementation of MAPs residues in RB, OR, BR and PP indicate that substrate enrichment in phenolics is able to influence *T. molitor* growth, while the beneficial effect of MAPs supplementation is reflected in the increased antioxidant activity of *T. molitor* reared in both RB and CC.

## Figures and Tables

**Table 1:** Proximate composition of the experimental by-products from agri-food industry used as substrates for *Tenebrio molitor* oviposition and rearing, supplemented with 0%, 10% or 20% distillation residues of Medicinal Aromatic Plants (MAPs).

Substrate	Dry Matter			Protein			Fat			Ash			Energy (MJ/kg)			Fiber and NFE		
	Addition of MAP (%)			Addition of MAP (%)			Addition of MAP (%)			Addition of MAP (%)			Addition of MAP (%)			Addition of MAP (%)		
	0	10	20	0	10	20	0	10	20	0	10	20	0	10	20	0	10	20
WB	86.0	88.7	88.4	21.5	19.8	19.7	5.4	5.2	6.1	5.6	5.0	5.1	19.1	19.4	19.7	67.5	70.0	69.0
RB	89.6	93.5	91.4	19.8	18.5	18.7	19.1	20.9	20.4	10.3	9.5	9.1	21.8	22.6	22.2	50.8	51.2	51.9
CC	88.1	91.0	90.5	7.3	5.9	6.2	2.2	1.7	2.3	1.5	1.9	2.8	18.3	18.6	18.9	88.9	90.5	88.8
OR	78.1	83.1	81.4	12.5	10.9	12.4	1.2	1.1	2.4	70.7	69.2	62.7	5.8	6.8	8.6	15.6	18.8	22.5
BR	90.4	89.1	89.7	25.3	24.3	22.0	2.0	2.4	2.5	27.3	24.8	24.9	14.4	15.6	15.4	45.4	48.4	50.5
PP	87.3	86.1	86.8	13.8	13.1	13.0	1.1	1.8	3.1	6.0	6.6	6.9	16.6	17.3	18.1	79.1	78.5	77.0

Abbreviations: WB, Wheat bran; RB, Rice bran; CC, Corn cob; OR, Olive oil residues; BR, Biogas residues; PP, Potato peel. % Mean of triplicate analysis on dry matter basis.

**Table 2:** Growth of *Tenebrio molitor* larvae fed agri-food industry by-products, supplemented with 0, 10% or 20% distillation residues of Medicinal Aromatic Plants (MAPs).

Substrate	Number of Larvae			Total Dry Weight		
	Addition of MAP (%)			Addition of MAP (%)		
	0	10	20	0	10	20
WB	37.0 ^ab^ ± 9.7b	82.0 ^a^ ± 14.6a	72.6 ^a^ ± 5.2ab	4.63 ^a^ ± 0.87a	6.49 ^a^ ± 0.67a	5.74 ^a^ ± 0.36a
RB	26.0 ^ab^ ± 5.2b	58.2 ^ab^ ± 9.4a	45.2 ^ab^ ± 13.2ab	0.68 ^b^ ± 0.11b	2.51^b^ ± 0.73a	2.02 ^b^ ± 0.43a
CC	48.6 ^a^ ± 9.7a	43.0 ^ab^ ± 13.1a	39.2 ^ab^ ± 9.8a	1.50 ^b^ ± 0.15a	1.73 ^bc^ ± 0.52a	1.40 ^bc^ ± 0.25a
OR	11.8 ^b^ ± 2.5b	35.0 ^b^ ± 6.6a	26.0 ^b^ ± 5.2ab	0.05 ^c^ ± 0.01b	0.32 ^c^ ± 0.02a	0.38 ^cd^ ± 0.09a
BR	12.0 ^b^ ± 0.5b	13.6 ^c^ ± 2.2ab	22.8 ^b^ ± 3.7a	0.05 ^c^ ± 0.01b	0.27 ^c^ ± 0.03a	0.29 ^d^ ± 0.02a
PP	25.0 ^ab^ ± 3.1a	44.8 ^ab^ ± 13.6a	28.0 ^b^ ± 4.4a	0.15 ^c^ ± 0.02b	0.62 ^bc^ ± 0.11a	0.43 ^cd^ ± 0.12ab

Abbreviations: WB, Wheat bran; RB, Rice bran; CC, Corn cob; OR, Olive oil residues; BR, Biogas residues; PP, Potato peel. Mean ± standard error, *n* = 5 independent replicates. Letters (a,b) indicate significant differences under the effect of different MAP ratio within the same substrate (horizontal basis comparisons), while superscript letters (a–d) following the mean values indicate significant differences under the effect of different substrate within the same MAP ratio, *p* < 0.05.

**Table 3:** Proximate composition of *Tenebrio molitor* larvae fed agri-food industry by-products supplemented with 0, 10% or 20% distillation residues of Medicinal Aromatic Plants (MAPs).

	**% MAPs**	**WB**	**RB**	**CC**	**OR**	**BR**	**PP**
Dry matter	0	39.3 ± 1.6 b	39.5 ± 1.1 b	31.6 ± 0.2 c	32.9 ± 0.6 c, A	33.3 ± 0.8 c	45.1 ± 1.3 a, A
	10	40.3 ± 0.6 ab	42.8 ± 0.2 a	30.6 ± 0.5 c	28.5 ± 2.1 c,B	31.9 ± 2.0 c	38.1 ± 0.3 b, B
	20	39.5 ± 1.1 ab	41.8 ± 0.2 a	31.6 ± 0.3 c	35.3 ± 1.8 b, A	30.1 ± 0.4 c	37.4 ± 0.0 ab, B
Protein (N x 6.25)	0	51.2 ± 0.9	53.4 ± 0.2	50.7 ± 0.6	57.5 ± 0.9 A	55.8 ± 2.0 B	59.5 ± 2.0 A
	10	52.1 ± 1.4 ab	47.7 ± 0.5 bc	51.3 ± 1.8 ac	56.0 ± 4.2 ab, A	61.7 ± 3.8 a, A	44.1 ± 2.0 c, B
	20	50.6 ± 0.1	47.5 ± 1.6	44.4 ± 4.1	42.8 ± 0.4 B	52.9 ± 6.5 B	43.6 ± 1.2 B
Protein (N x 4.76)	0	39.0 ± 0.7	40.7 ± 0.1	38.6 ± 0.4	43.8 ± 0.7 A	42.5 ± 1.5 B	45.3 ± 1.5 A
	10	39.7 ± 1.1 ab	36.4 ± 0.4 bc	39.1 ± 1.3 ac	42.7 ± 3.2 ab, A	47.0 ± 2.9 a, A	33.6 ± 1.5 c, B
	20	38.5 ± 0.1	36.2 ± 1.2	33.8 ± 3.1	32.6 ± 0.3 B	40.3 ± 4.9 B	33.2 ± 0.9 B
Fat	0	25.2 ± 2.5 b	31.3 ± 0.3 a	16.6 ± 1.1 c	23.9 ± 1.6 b, A	20.9 ± 0.5 bc	21.7 ± 2.2 bc, B
	10	26.6 ± 0.6 ab	30.7 ± 0.3 ab	15.5 ± 0.7 c	10.4 ± 0.4 c, B	22.2 ± 0.7 b	29.6 ± 1.4 a, A
	20	28.2 ± 3.1 a	31.3 ± 0.2 a	16.0 ± 0.1 bc	11.5 ± 0.6 c, B	17.8 ± 1.6 bc	29.7 ± 0.9 a, A
Ash	0	5.5 ± 0.2 b	5.0 ± 0.4 b	5.3 ± 0.1 b, A	6.7 ± 0.2 b, C	10.2 ± 0.1 a, A	10.3 ± 0.5 a, A
	10	4.8 ± 0.2 c	5.3 ± 0.3 d	2.5 ± 0.1 e, B	9.8 ± 0.4 a, B	7.7 ± 0.2 b, B	5.4 ± 0.9 c, B
	20	5.2 ± 0.4 d	6.1 ± 0.4 bc	5.2 ± 0.3 c, A	13.1 ± 0.7 a, A	7.8 ± 0.5 b, B	5.3 ± 0.4 c, B
Fiber & NFE	0	18.1 ± 3.2 ab	10.3 ± 0.8 b	27.5 ± 1.6 a	11.9 ± 1.0 b, B	13.2 ± 2.2 b, AB	8.5 ± 3.8 b, B
	10	16.4 ± 2.1 bc	16.2 ± 0.8 bc	30.7 ± 2.5 a	23.7 ± 3.5 ab, A	8.4 ± 4.1 c, B	20.9 ± 1.6 bc, A
	20	16.0 ± 2.7 c	15.1 ± 1.0 c	34.4 ± 3.8 a	32.6 ± 1.7 ab, A	21.5 ± 6.7 bc, A	21.4 ± 0.5 c, A
**Two-way ANOVA *p*-values**							
		**Substate type**		**MAP addition**		**Substrate x MAP**	
Dry matter		<0.001		0.046		<0.001	
Protein		0.004		<0.001		0.005	
Fat		<0.001		Non-significant		<0.001	
Ash		<0.001		<0.001		<0.001	
Fiber & NFE		<0.001		<0.001		<0.010	

Fiber and NFE (nitrogen-free extract) = 100% − % protein (N x 6.25) − % fat − % ash. Abbreviations: WB: Wheat bran; RB: Rice bran; CC: Corn cob; OR: Olive oil residues; BR: Biogas residues; PP: Potato peel. Different letters (a–e) indicate significant differences under the effect of different substrate within the same MAP ratio (horizontal basis comparisons), while different capital letters (A–C) indicate significant differences under the effect of different MAP ratio within the same substrate (vertical basis comparisons).

**Table 4:** Correlation analysis between the substrates’ proximate composition and the characteristics of *Tenebrio molitor* larvae.

	no. of TML		Total Dry Larval Weight		TML Dry Matter		TML Protein		TML Fat		TML Ash		TML Fiber & NFE	
Substrates’	r	Sig.	r	Sig.	r	Sig.	r	Sig.	r	Sig.	r	Sig.	r	Sig.
Dry matter	0.270	NS	0.304	NS	0.112	NS	−0.049	NS	0.228	NS	−0.397	NS	−0.067	NS
Protein	−0.252	NS	−0.069	NS	0.333	NS	0.368	NS	0.439	NS	0.222	NS	−0.657	**
Fat	0.427	NS	0.636	**	0.496	*	−0.377	NS	0.684	**	−0.368	NS	−0.162	NS
Ash	−0.682	**	−0.671	**	−0.164	NS	0.315	NS	−0.051	NS	0.699	***	−0.302	NS
Energy	0.727	***	0.843	***	0.591	**	−0.362	NS	0.643	**	−0.703	***	−0.090	NS
Fiber and NFE	0.561	NS	0.483	*	0.139	NS	−0.354	NS	0.046	NS	−0.602	**	0.323	NS

Abbreviations: no., number; NFE, nitrogen-free extract; sig., significance; WB, Wheat bran; RB, Rice bran; CC, Corn cob; OR, Olive oil residues; BR, Biogas residues; PP, Potato peel. Notes: Correlation analysis was conducted for all substrates collectively, including the three levels of 0, 10 and 20% incorporation of MAPs. Significant correlations are presented with asterisks for *p* ≤ 0.001 ***, *p* ≤ 0.01 ** and *p* < 0.05 *. NS, non-significant correlations.

**Table 5:** Fatty acids composition of *Tenebrio molitor* larvae (% of total fatty acids) fed by-products from agri-food industry supplemented with 0, 10% or 20% distillation residues of Medicinal Aromatic Plants (MAPs).

Fatty Acid	% MAPs	WB	CC	RB	OR	BG	PP	SEM_pooled_
C10:0	0	0.26 f, B	0.66 d, B	0.36 e, A	0.85 c, B	1.31 b, A	2.62 a, A	0.01
	10	0.38 e, A	0.64 b, C	0.21 f, B	0.61 c, C	0.87 a, C	0.44 d, C	
	20	0.23 f, C	0.71 c, A	0.35 e, A	1.14 a, A	0.89 b, B	0.46 d, B	
C14:0	0	3.62 e, B	4.72 b, A	2.78 f, C	4.10 d, B	5.09 a, A	4.13 c, A	0.01
	10	4.11 b, A	4.63 a, B	3.73 f, A	4.02 d, C	4.11 c, B	3.99 e, B	
	20	3.14 e, C	4.12 b, C	2.89 f, B	4.99 a, A	3.57 d, C	3.81 c, C	
C16:0	0	11.0 f, B	14.4 c, A	12.1 d, B	16.9 a, A	16.2 b, A	16.3 b, A	0.03
	10	11.7 e, A	13.1 cd, B	13.0 d, A	13.7 a, B	13.4 bc, B	13.5 ab, B	
	20	6.76 f, C	13.0 a, B	10.1 d, C	11.9 c, C	12.2 b, C	8.85 a, C	
C16:1	0	2.18 e, C	2.84 d, A	1.36 f, C	2.94 c, A	3.10 b, A	3.57 a, A	0.11
	10	2.95 b, A	2.50 de, C	2.77 c, A	2.51 d, B	1.92 e, C	3.47 a, C	
	20	2.66 b, B	2.56 c, B	1.81 f, B	2.15 e, C	2.21 d, B	3.54 a, B	
C17:0	0	1.30 e, B	3.13 d, C	1.20 f, C	6.37 c, A	8.09 a, A	6.81 b, A	0.02
	10	1.91 d, A	4.47 b, A	1.29 e, A	1.94 d, C	5.36 a, C	2.14 c, B	
	20	1.25 e, C	3.32 c, B	1.24 e, B	5.76 b, B	6.35 a, B	1.36 d, C	
C18:0	0	2.48 e, A	3.75 c, A	3.62 c, A	3.35 d, C	5.05 b, A	6.68 a, A	0.08
	10	2.17 e, B	3.38 c, B	2.44 d, C	3.58 b, A	3.88 a, B	2.20 e, B	
	20	1.16 d, C	3.20 a, C	2.70 b, B	3.35 a, B	3.36 a, C	1.51 c, C	
C18:1	0	47.4 a, B	46.0 b, A	44.8 c, C	46.9 a, A	41.8 d, B	38.4 e, C	0.28
	10	45.8 d, C	42.3 e, C	48.7 b, B	46.6 c, A	39.3 f, C	52.5 a, B	
	20	49.6 b, A	45.0 c, B	49.4 b, A	42.1 e, B	43.0 d, A	56.0 a, A	
C18:2 ω6	0	27.6 b, B	19.1 c, C	28.8 a, A	15.6 d, C	15.0 e, C	14.0 f, C	0.05
	10	26.1 a, C	18.8 d, A	25.3 b, C	20.7 c, A	18.5 e, A	15.5 f, B	
	20	30.0 a, A	20.1 c, B	28.4 b, B	18.0 d, B	18.0 d, B	17.6 e, A	
C20:1	0	0.57 a, C	0.20 e, C	0.33 d, C	0.38 bc, C	0.41 b, C	0.34 cd, C	0.02
	10	0.75 e, B	2.18 b, B	0.62 f, A	1.03 d, B	8.30 a, A	1.65 c, B	
	20	0.94 d, A	2.36 b, A	0.57 e, B	2.09 c, A	4.85 a, B	2.34 b, A	
SFAs	0	18.9 e, B	27.0 c, A	20.2 d, B	31.8 b, A	36.6 a, A	37.0 a, A	0.20
	10	20.6 e, A	26.9 b, A	20.9 e, A	24.3 c, C	27.8 a, B	22.7 d, B	
	20	12.9 f, C	24.9 c, B	17.5 d, C	27.6 a, B	26.8 b, C	16.5 e, C	
MUFAs	0	50.2 a, B	49.4 b, B	46.5 c, C	50.7 a, A	46.6 c, C	44.0 d, B	0.25
	10	49.9 cd, B	47.3 e, C	52.4 b, A	50.5 c, A	49.6 d, B	58.1 a, A	
	20	53.6 b, A	50.3 d, A	52.0 c, B	46.3 e, B	50.4 d, A	62.3 a, A	
PUFAs	0	27.8 b, B	19.4 c, B	29.1 a, A	15.8 e, C	16.1 d, C	14.4 f, C	0.06
	10	26.7 a, C	18.8 d, C	25.6 b, B	21.3 c, A	18.5 e, A	15.9 f, B	
	20	30.5 a, A	20.5 c, A	29.0 b, A	18.5 d, B	18.4 d, B	18.0 e, A	
Omega 6	0	27.7 b, B	19.3 c, B	29.0 a, A	15.7 d, C	15.7 d, C	14.2 e, C	0.06
	10	26.3 a, C	18.8 d, C	25.4 b, C	21.1 c, A	18.5 e, A	15.7 f, B	
	20	30.2 a, A	20.4 c, A	28.8 b, B	18.2 d, B	18.2 d, B	17.9 e, A	

Abbreviations: SFAs, saturated fatty acids; MUFAs, mono-unsaturated fatty acids; PUFAs, poly-unsaturated fatty acids; WB, Wheat bran; RB, Rice bran; CC, Corn cob; OR, Olive oil residues; BR, Biogas residues; PP, Potato peel Mean ± pooled standard error of the mean. Notes: Two-way ANOVA: substrate *p* < 0.001; MAP inclusion *p* < 0.001; substrate x MAP inclusion *p* < 0.001 for all the fatty acids presented here. In this table, individual fatty acids with maximum value < 0.5% are not included. Different letters (a–f) indicate significant differences under the effect of different substrate within the same MAP ratio (horizontal basis comparisons), while capital letters (A–C) indicate significant differences under the effect of different MAP ratio within the same substrate (vertical basis comparisons), *p* < 0.05.

**Table 6:** Correlation analysis between the developmental characteristics of *Tenebrio molitor* larvae and their content in total phenols, total flavonoids and antioxidant potential measured with two assays (ABTS and FRAP).

Substrate	N	No. of Larvae x Total Weight		Total Weight x Phenols		Total Weight x Flavonoids		Total Weight x ABTS		Total Weight x FRAP	
		r	Sig.	r	Sig.	r	Sig.	r	Sig.	r	Sig.
WB	9	0.915	**	−0.52	NS	0.761	*	−0.719	*	−0.387	NS
RB	9	0.896	**	0.293	NS	0.166	NS	0.646	NS	0.766	*
CC	9	0.578	*	0.031	NS	−0.131	NS	−0.402	NS	0.278	NS
OR	9	0.444	NS	0.910	**	0.911	**	0.916	**	0.786	**
BR	9	0.177	NS	0.359	NS	0.681	*	0.773	*	0.470	NS
PP	9	0.404	NS	0.271	NS	0.163	NS	0.341	NS	0.312	NS
All substrates	54	0.814	**	−0.016	NS	−0.416	**	−0.584	**	0.980	NS
Basic substrate	17	0.571	*	0.649	**	−0.374	NS	0.371	NS	0.393	NS

Abbreviations: no., number; significance, sig.; WB, Wheat bran; RB, Rice bran; CC, Corn cob; OR, Olive oil residues; BR, Biogas residues; PP, Potato peel. Notes: Correlation analysis was conducted for each different substrate (including the three levels 0,10% and 20% incorporation of MAPs individually). Significant correlations are presented with asterisks for *p* ≤ 0.01 ** and *p* < 0.05 *. NS, non-significant correlations.

**Table 7:** Correlation analysis of the content in total phenols, total flavonoids and antioxidant potential measured with two assays (ABTS and FRAP) between the TML and the substrates they were fed with.

Substrate	N	Correlation between Substrates Contents x Larvae Contents							
		Total Phenols		Total Flavonoids		ABTS		FRAP	
		r	Significance	r	Significance	r	Significance	r	Significance
WB	9	−0.673	*	0.283	NS	−0.039	NS	−0.226	NS
RB	9	0.385	NS	0.087	NS	0.833	*	0.786	*
CC	9	0.922	**	0.169	NS	0.550	NS	0.922	**
OR	9	0.951	**	0.819	**	0.851	**	0.943	**
BR	9	0.362	NS	0.841	**	0.786	**	0.579	NS
PP	9	0.690	*	−0.352	NS	0.738	*	−0.436	NS
All substrates	54	0.417	**	0.504	**	0.357	**	0.641	**
Basic substrate	17	0.450	NS	0.460	NS	0.353	NS	0.069	NS

Abbreviations: WB, Wheat bran; RB, Rice bran; CC, Corn cob; OR, Olive oil residues; BR, Biogas residues; PP, Potato peel. Notes: Correlation analysis was conducted for each different substrate (including the three levels 0,10% and 20% incorporation of MAPs individually). Significant correlations are presented with asterisks for *p* ≤ 0.01 ** and *p* < 0.05 *. NS, non-significant correlations.

## Data Availability

All of the data is contained within the article and Supplementary Materials.

## Supplementary Materials

The following are available online at https://www.mdpi.com/article/10.3390/antiox11010068/s1, Figure S1: Representative gas chromatograms of fatty acid methyl esters derived from *Tenebrio molitor* larvae fed the different basic substrates (wheat bran, rice bran, corn cob, olive oil residues, biogas residues, potato peel).
