# Supplementary material for: Towards Functional Insect Feeds: Agri-Food By-Products Enriched with Post-Distillation Residues of Medicinal Aromatic Plants in Tenebrio molitor (Coleoptera: Tenebrionidae) Breeding
Source: Antioxidants (Basel). 2021 Dec 28;11(1):68. doi: 10.3390/antiox11010068 (PMC8772721; doi:10.3390/antiox11010068)
Supplement: Supplementary file 1 [file antioxidants-11-00068-s001.zip › antioxidants-1518469-supplementary.pdf]

antioxidants

MDPI

Article

Towards Functional Insect Feeds: Agri-Food By-Products Enriched with Post-Distillation Residues of Medicinal Aromatic Plants in *Tenebrio molitor* (Coleoptera: Tenebrionidae) Breeding

Stefanos S. Andreadis [1,*], Nikolas Panteli [2,†], Maria Mastoraki [2,3,†], Eleftheria Rizou [1,†], Vassilia Stefanou [2], Sofia Tzentilasvili [2], Eirini Sarrou [1], Stavros Chatzifotis [3], Nikos Krigas [1] and Efthimia Antonopoulou [2,*]

[1] Institute of Plant Breeding and Genetic Resources, Hellenic Agricultural Organization—Demeter, 57001 Thermi, Greece; elef.rz@gmail.com (E.R.); esarroy@gmail.com (E.S.); nikoskrigas@gmail.com (N.K.)
[2] Department of Zoology, School of Biology, Aristotle University of Thessaloniki, 54124 Thessaloniki, Greece; nkpanteli@bio.auth.gr (N.P.); mmastora@bio.auth.gr (M.M.); vasiliastefanou@hotmail.com (V.S.); tzentila@bio.auth.gr (S.T.)
[3] Institute of Marine Biology, Biotechnology and Aquaculture, Hellenic Centre for Marine Research, Gournes Pediados, 71003 Heraklion, Greece; stavros@hcmr.gr
* Correspondence: stefandr@ipgrb.gr (S.S.A.); eantono@bio.auth.gr (E.A.); Tel.: +30-2310-471110 (S.A.); +30-2310-998563 (E.A.)
† Authors contributing equally.

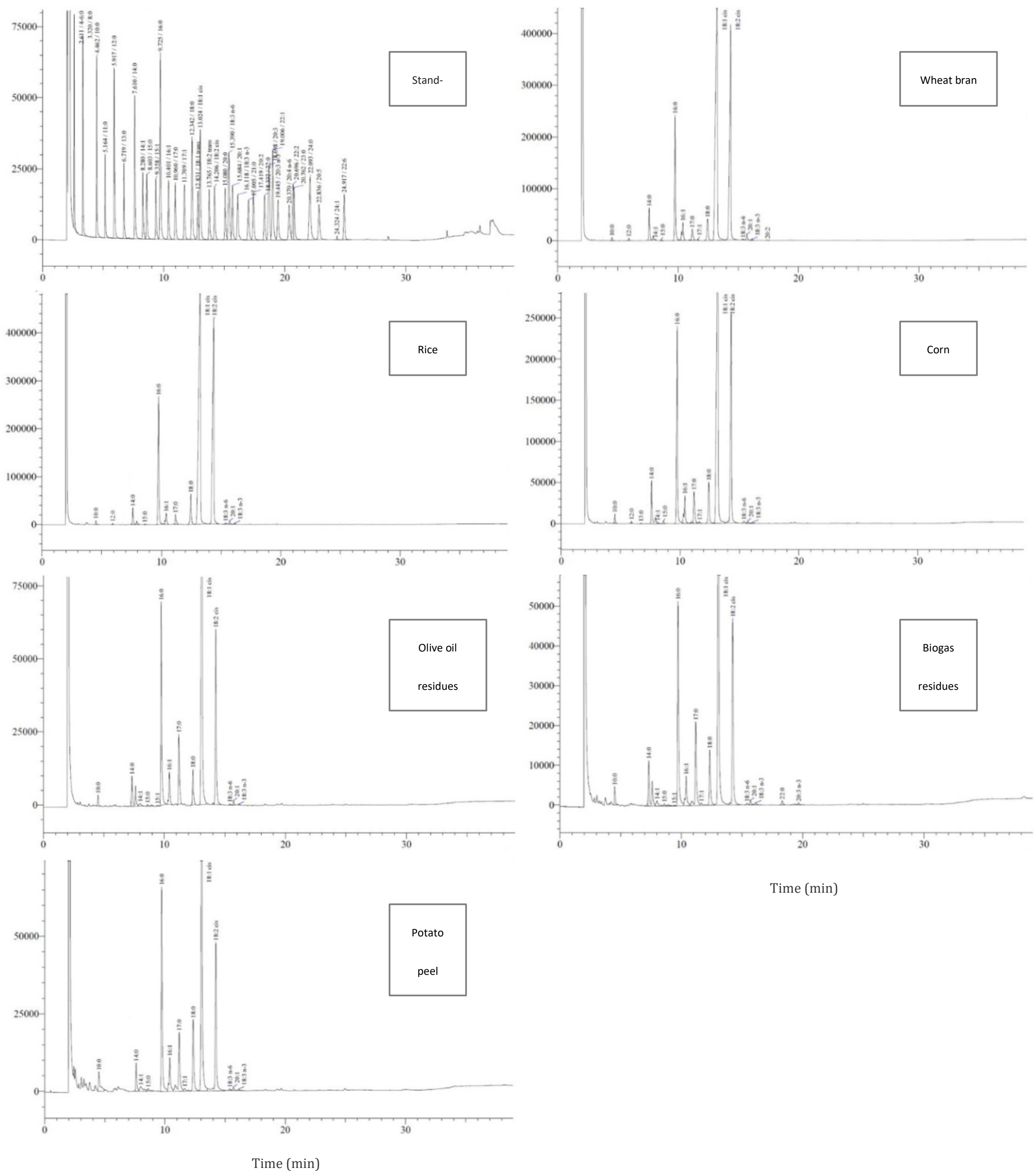

Figure S1. Representative gas chromatograms of fatty acid methyl esters derived from *Tenebrio molitor* larvae fed the different basic substrates (wheat bran, rice bran, corn cob, olive oil residues, biogas residues, potato peel).
